# The impact of COVID-19 pandemic on medicine security in Africa: Nigeria as a case study

**DOI:** 10.11604/pamj.supp.2020.35.2.23671

**Published:** 2020-06-10

**Authors:** Wuraola Akande-Sholabi, Yusuff Adebayo Adebisi

**Affiliations:** 1 Department of Clinical Pharmacy and Pharmacy Administration, Faculty of Pharmacy, University of Ibadan, Ibadan, Nigeria

**Keywords:** Pharmaceuticals, access to medicines, medicine security, COVID-19 pandemic, Nigeria, Africa

## Abstract

COVID-19 is an unprecedented pandemic posing major threat to global public health. In the past decades of years or so, one could have heard of how dangerous it is to be virtually reliant on medicine supply from other countries. Nonetheless, no action was taken because it seemed to many that the global trade system was operational and Nigerians as well as citizens of African countries appear to have sufficient supply of the medications required at quite appealing cost. Currently in 2020, this apprehension has revolved from an imaginary problem to an actual challenge that might have consequences for millions nationwide due to COVID-19 pandemic. Now, African countries can realize that putting all our eggs in one basket was not such a good idea. In Nigeria, over 70% of the prescribed medications are produced from active ingredients (API) primarily sourced from firms in China and India. Access to medicine is an integral part of healthcare systems, uninterrupted access to medicine is much needed and essential for the well-being of the population. We are now approaching the conclusion that it is more reasonable to probably invest a little more to resuscitate a domestic pharmaceutical synthesis and herbal medicine research capacity in Nigeria and across African countries to improve public health.

## Commentary

Africa is the world´s second-most populous continent, after Asia. Africa is made up of 54 countries, and Nigeria is the most populous country in Africa. Many African countries face double burden of communicable and non-communicable diseases and Nigeria is not an exception [1]. The country is also not exempted from the health threat imposed by COVID-19 pandemic [2]. Coronavirus disease 2019 (COVID-19), the disease caused by the Severe Acute Respiratory Syndrome Coronavirus 2 (SARS-CoV-2), was first seen in China where it became so widespread that numerous manufacturing factories and distribution services were incapacitated due to employee illness and absence initiating closures from a few weeks to several months. Nigeria has reported 5445 confirmed cases, discharged 1320, and 171 deaths in 34 states and the Federal Capital Territory, as of 15 May 2020 [3]. The Nigerian government has mounted responses causing against the pandemic to further reduce the spread of the virus and treat infected people. Even though the country is facing various health issues, the central attention is likely to be primarily towards effective containment of this unprecedented pandemic. The country´s responses to the pandemic is expected to have impacts on healthcare system and medicine security is not an exception. Access to medicine is an important component of good health systems and with uninterrupted access to medicine, improving the health outcome of the population is likely to be achieved. Nigeria is blessed with over 200 million population and the health issues in this resource-limited setting is on the rise and the need to respond to the pandemic remains imperative.

Despite limited resources, national health authorities have been making efforts to ensure effective containment of the pandemic. However, what remains a concern is that as the health threat of the pandemic continues to grow, other health issues and related interventions and programs are likely to be deprioritized. Currently, some of the issues facing responses to COVID-19 pandemic in many African countries including Nigeria are limited conducive isolation centres, diagnostic insufficiency, violation of the stay-at-home order, limited hospital capacity, congested cities, and family clustering among others [1]. As the stakeholders continues to address these issues, it is important that other health-related needs are prioritized including uninterrupted access to medicines during this pandemic. Nigeria is highly dependent on other countries for its medicine needs. About 70% of the medicines use in Nigeria and most other African countries are imported from China and India [4]. The country also relies on other countries for active pharmaceutical ingredients, equipment and other resources needed for medicine manufacturing [4-6]. As COVID-19 confirmed cases and mortality continue to rise, this dependency on foreign countries for medicines, API and other needed resources for drug manufacturing remain a major concern. Although the local drug industry has experienced some growth over the years, the country still falls short in local drug manufacturing for its needs.

One of the keyways to ensure improvement of medicine security in Nigeria is to address the challenges facing medicines manufacturing at the country level, as well as those hampering effective supply chain management of medicines. The ban on the international travel during this pandemic may pose a serious challenge to the healthcare system in Nigeria and across Africa [1] because of heavy reliance of Nigeria on importation from other countries. The big questions amidst this pandemic still remains (1) Will the local pharmaceutical industry in Nigeria be able to cater for the medicine needs? (2) Will the international travel ban obstruct the availability of essential medicines in Nigeria? It is glaringly obvious that the outbreak of COVID-19 is ushering a dynamic era of approach to strengthen our health care system and the stakeholders need to take up their role to seize this opportunity to improve in-country drug manufacturing in Nigeria. Disruption in access to medicines in Nigeria remains a threat to United Nations´ Sustainable Development Goals especially goal 1, 3, 8 and 9, which seeks an end to extreme poverty, ensure good health and well-being, promote sustained, inclusive economic growth and productivity, as well as sustainable industrialisation. Boosting the pharmaceutical local drug manufacturing can contribute to the country´s GDP by exporting some of the locally made herbal remedies to other countries. This implies investing in in-country drug manufacturing can have impacts on the general well-being of the population. Unfortunately, since the establishment of the National Drug Policy in 2005, the growth established for the implementation has been crawling. This pandemic further reiterated the need for local drug manufacturing in Nigeria and across Africa especially due to the condition the world is in, where importation is nearly impossible.

In-country drug manufacturing is crucial in combating diseases, reducing mortality rate and catering for other medicine-related healthcare needs toward improving general well-being and health of the population. Expanding local drug manufacturing is also another opportunity to combat falsification and trade of substandard and counterfeit pharmaceutical products in the country. Additionally, Nigeria can offset dependency on imports to meet its medicines need by improving its manufacturing capacity, increasing research capacity, funding local research in seeking for medicines from plant and encouraging the use of its locally made medicines in management of different ailments. It is imperative to address challenges facing the pharmaceutical sectors, such as infrastructural problems, dearth of pharmaceutical scientists, weak technology and engineering base, industrial linkages, and supply chain challenge among others. Compared to many other African countries, Nigeria has a relatively sizeable pharmaceutical industry over 100 pharmaceutical companies [7,8]. With the existing fiscal and monetary policies, most of the pharmaceutical companies in Nigeria are still having challenges in addressing these obstacles resulting into limited in-country medicine production for its population need [4].

## Conclusion

The net impact of COVID-19 pandemic may occur soon with worrisome outcome on the health of Nigerians. In addition, the threat to medicine insecurity due to the pandemic could result into increase in circulation of fake and counterfeit medicines putting lives of millions at danger. This is a further concern in that the country has been struggling over the years to fight fake, counterfeited, and sub-standard medicines in its supply chain. It is also very important for the country to fund herbal medicine research and increase its commitment towards advancing it. Extensive research should be implored to fully utilize the potential of herbal medicine because Nigeria is blessed with thousands of plants with potential health benefits. Furthermore, the need to encourage local pharmaceutical companies and herbal medicine research is essential towards improving public health.

## Competing interests

The authors declare no competing interests.

## Authors’ contributions

The concept for this commentary was developed by Wuraola Akande-Sholabi. Yusuff Adebayo Adebisi and Wuraola Akande-Sholabi developed the draft and prepared the manuscript. All the authors have read and agreed to the final manuscript.
